# Endogenous Intoxication and Saliva Lipid Peroxidation in Patients with Lung Cancer

**DOI:** 10.3390/diagnostics6040039

**Published:** 2016-11-16

**Authors:** Lyudmila V. Bel’skaya, Victor K. Kosenok, Gilbert Massard

**Affiliations:** 1 KhimServis Limited Liability Company, 143026 Moscow, Russia; vic.kos_senok@mail.ru; 2 Chair of Chemical Technology and Biotechnology, Omsk State Technical University, 644050 Omsk, Russia; 3 Chair of Oncology with Radiotherapy, Omsk State Medical Academy, 644099 Omsk, Russia; 4 Strasbourg University Hospital, 67000 Strasbourg, France; gilbert.massard@chru-strasbourg.fr

**Keywords:** saliva, lung cancer, lipid peroxidation, middle molecules, malondialdehyde

## Abstract

This research was aimed at a search for regularities in changes to parameters of endogenous intoxication and saliva lipid peroxidation in patients with lung cancer, non-malignant lung diseases, and apparently healthy people. All patients went through saliva sampling at an amount of 1 mL. A concentration of malondialdehyde (MDA) was measured according to a reaction with thiobarbituric acid, and a level of middle molecules (MM) was measured with UV spectroscopy at 254 and 280 nm, while the content of lipid peroxidation products was measured according to a degree of heptane extract light absorption at wavelengths of 220, 232, 278, and 400 nm. It has been revealed that in the context of lung cancer, the level of diene conjugates decreases, increasing the level of triene conjugates, Schiff’s bases, and MM. As a tumor grows, there is a decrease in the level of lipid peroxidation primary products and an increase in endotoxemia phenomena. The process is more apparent when going from local to locally advanced disease states. The nature of the MDA change is nonlinearly associated with tumor progression. The findings might be used to optimize traditional aids of diagnostics, in disease state forecasting, in treatment monitoring, etc.

## 1. Introduction

The elaboration of aids in diagnostics and forecasting of lung cancer course, a top oncologic disease [1,2,3,4], is still a priority. An excessive formation of reactive oxygen species, which, besides their evident mutagenic effect, initiate lipid peroxidation processes in biological membranes, is a crucial factor involved in the mechanisms of carcinogenesis. The oxidative stress that occurs in a body is considered a key process in changes to programs of cell differentiation, proliferation, and apoptosis. It is associated with reactive oxygen species that damage mitochondrial DNA, which leads to bioenergetic crisis and a release of apoptotic protease activating factors into the cytoplasm [5,6]. Free radicals are involved in all stages of tumor development, starting from the moment of malignant transformation in cells. They contribute to tumor growth and its invasive and metastatic potential, and trigger a cascade of reactions that lead to irreversible consequences in the cell [7]. In this context, endogenous intoxication occurs accompanied by the increased level of middle molecules (MM).

MM affect the functioning of all body systems and organs. Their structure is similar to that of regulatory peptides [8]. They can connect and block cell receptors, thereby changing intracellular metabolism and functions [9]. This is particularly evident in inflammatory processes of various localizations, in case of an exposure to toxic chemicals, and in malignant tumor growth [9,10,11]. Endogenous intoxication substrates include reactive oxygen species; products of lipid peroxidation and oxidative modification of proteins; peroxides, hydroperoxides, diene conjugates, and malondialdehyde.

Parameters of endogenous intoxication, as well as products of lipid peroxidation, are traditionally measured in blood serum and plasma, but it is also possible to use mixed saliva as a substrate [12]. Saliva research using numerous clinical and biochemical parameters has its advantages compared to routine laboratory diagnostics using venous or fingertip blood samples [13,14]. The advantages are the following: simple and convenient saliva sampling, a noninvasive and painless procedure, no infection risk when a biomaterial is taken. At the same time, the saliva adequately reflects the biochemical status and the physiological state of a person. This biomaterial is widely used, i.e., in humans, in hygiene and toxicology research, in the research of drug pharmacodynamics and for special scientific purposes [15,16].

Processes of lipid peroxidation are crucial in the pathogenesis of endogenous intoxication. A number of papers deal with the research of these processes in saliva [17], but most papers are about an evaluation of the level of the end product, i.e., malondialdehyde [18,19], as well as the level of antioxidant enzymes [20,21]. The following papers are focused on the problem of periodontal disease along with clinically significant somatic pathologies, in particular hypertension, ischemic heart disease, epilepsy, diabetes, etc. Special attention has been given to the comparison of saliva under normal conditions and that in smokers [22]. The researchers have reported changes to processes of lipid peroxidation in the saliva of patients with oral pre-cancer and cancer as exemplified by malondialdehyde [23,24,25,26]. However, the possibility of using saliva as a material to study the processes of lipid peroxidation in patients with lung cancer has not been considered so far.

This research was aimed at a search for regularities in changes to endogenous intoxication parameters and saliva lipid peroxidation in patients with lung cancer, nonmalignant lung diseases and apparently healthy people to elaborate traditional prognosis and diagnostic aids for the disease course.

## 2. Materials and Methods

The research included 445 patients from the Omsk Clinical Oncologic Dispensary (29% female, 71% male), and 535 apparently healthy persons, selected as a control group. The average age of patients was 58.5 ± 0.9 years for men, 56.0 ± 2.1 years for women, and 49.4 ± 4.7 years for the control group. Differences between the groups are statistically significant (*p* < 0.001), therefore in the course of research the groups were divided into subgroups at intervals of 10 years: 40–49, 50–59, 60–69, and over 70. The study group included 337 patients with lung cancer of different histologic types (squamous cell carcinoma, small-cell carcinoma, and adenocarcinoma) and of various growth form (central and peripheral). 108 patients had a nonmalignant lung disease, of these seven had chronic pneumonia, 20 had pulmonary tuberculosis, 28 persons had hamartoma, 10 persons had sarcoidosis, and 24 people had fibrosis, etc. Healthy controls were selected among individuals that had not have a history of mucosal diseases in the oral cavity, immunodeficiency, autoimmune disorders, hepatitis, or HIV infection, Parkinson’s disease. Study groups were made according to clinical trials regulations upon receiving the informed consent. The inclusion criteria were the following: patients’ age of 30–75, no treatment in time of the survey (including surgery, chemotherapy, or radiation), no signs of an active infection (including purulent processes), and oral cavity sanation.

Saliva samples were collected from two groups between 8 and 10 a.m., at least 2 h after their last meal and oral hygiene to rule out the influence of circadian rhythm and effects of diet and hygiene products on salivary composition. Saliva samples were collected with saliva spitting into sterile test-tubes. Collection was continued until 1 mL of saliva was obtained, or the collection was 10 min long. The saliva samples were centrifuged at 7000 rpm for 10 min. All tests were carried out in duplicate or in triplicate if the results could not be reproduced. All samples were evaluated for the level of acyl hydroperoxides. To do that, 0.200 mL of saliva were placed in a separate tube, where 4 mL of heptane-isopropanol mixture (1:1) were added, and the contents were shaken for 10–15 min. Then, 1 mL of HCl solution (рН 2) and 2 mL of heptane were added, the mixture was then intensively shaken and allowed to stand for 20–25 min. Out of this mixture, split into phases, the upper heptane layer was taken to measure the concentration of acyl hydroperoxides by degree of light absorption at the wavelengths of 220, 232, 278, and 400 nm. It should be mentioned that light absorption at the wavelength of 220 nm (E220) corresponds to absorption of isolated double bonds. Light absorption at the wavelength of 232 nm (E232) corresponds to absorption of diene conjugates; light absorption at the wavelength of 278 nm (E278) corresponds to absorption of triene conjugates; absorption at the wavelength of 400 nm (E400) corresponds to absorption of Schiff’s bases. To increase precision of measurements and eliminate indicated errors, ratios of E232/E220, E278/E220, and E400/E220 were calculated; levels of lipid peroxidation products were expressed with relative units, namely, diene conjugates as E232/E220, triene conjugates as Е278/Е220, and Schiff’s bases as Е400/Е220.

To measure the level of middle molecules, 0.2 mL of 16% trichloroacetic acid solution (TCA) were added to 0.200 mL of saliva, then proteins were spinned down by centrifuging for 10 min at 200 rpm. Then, 2.8 mL of the distilled water were added to 0.2 mL of supernatant and then UV-absorption was measured with a spectrophotometer at 254 and 280 nm in quartz cells, with the path length of 1 cm versus the blank sample. In the blank sample, 0.1 mL of the stock solution of TCA was added to 2.9 mL of the water. MM levels were expressed in unites, which were quantitatively equal to extinction indices. Moreover, the value of the partition coefficient (middle molecules at 280/254 nm) was calculated as a ratio of extinctions at the wavelengths of 280 and 254, respectively.

The method to measure malondialdehyde (MDA) is based on the fact that at high temperatures in an acidic medium, MDA reacts with thiobarbituric acid, making the colored pink trimethine complex with the highest absorption at 535 nm. 2 mL of the distilled water and 1 mL of 0.6% TBA in glacial acetic acid were added to 0.200 mL of saliva. The solution was boiled for 30 min, cooled, then 1 mL of 5% potassium hydroxide and 2 mL of isopropanol were added. The solution was centrifuged at 6000 rpm for 20 min, and measured at 535 and 580 nm versus the control sample, containing the water instead of the saliva.

The statistical analysis of findings was made with the Statistica 10.0 (StatSoft) software and R Package (version 3.2.3), using the nonparametric method, applying the Wilcoxon test for dependent groups and the Mann–Whitney U test for independent groups.

## 3. Results

### 3.1. Measuring Levels of Middle Molecules

MM are substances with a molecular weight ranging from 1500 to 5000 D which are universal markers of endotoxemia [8]. The nature of MM is inhomogeneous. It has been found that most chemically identified MM are fragments of endogenous proteins. Perhaps acyl hydroperoxides and fragments of damaged cell membranes are primary products that make MM. Being natural biogenic regulators under normal conditions, when in high concentrations, MM have a wide range of pathological effects. MM inhibit mitochondrial respiration, DNA synthesis in alveolar macrophages and lymphocytes, and hemoglobin synthesis; they also reduce lactate dehydrogenase activity, etc. [27].

The ultraviolet spectrophotometry of biologic fluids is a basic method to measure MM levels [9]. The wavelength range of 220–255 nm is the peak absorption spectrum of non-aromatic (254 nm) sulphur-containing amino acids (cystine, cysteine, and methionine); 250–256 nm is the maximum of extinctions for purine bases (adenine, guanine). Substances of a catabolic origin, xenobiotics, cellular and tissue debris, and microbial substances are registered in the wavelength range of 238–242 nm. The wavelength range of 238–244 nm is the peak absorption spectrum for urea, uric acid, and creatinine. The degradation product absorption spectra of albumin, fibrinogen, non-esterified fatty acids, and other substances are close to this maximum. High values of extinctions at the wavelengths of 238, 242, and 246 nm always point to pathologic processes in the body [27]. At the wavelength of 258 nm, there is the peak absorption of adenosine diphosphate, adenosine monophosphate, adenine, l-valine, and l-phenylalanine, while at the wavelength of 280 nm, the light is maximally absorbed by aromatic chromophores, i.e., phenols, tyrosine, tryptophan, and phenylalanine.

At the first research stage, MM levels at the wavelengths of 254 nm and 280 nm were measured in the saliva of apparently healthy people (Table 1), and in that of patients with lung diseases (Table 2).

According to the findings (Table 1), the absolute values of extinctions at the wavelengths of 254 and 280 nm in the saliva of apparently healthy patients are higher for men than for women. However, MM partition coefficients at 280/254 nm in both groups are similar. Statistically significant differences have been only found for the age groups of 60–69 years and over 70 years. It should also be also that an increase in the partition coefficient under normal conditions when going from the youngest age group (40–49 years) to the oldest (over 70 years) amounts to 14% for men, and 2.7% for women. This means that under normal conditions, age endotoxemia phenomena are more apparent in men than in women.

In the context of lung cancer, absolute values of extinctions at the explored wavelengths for men and women are close for the age groups of 50–59 and 60–69 years. For the younger and the older age groups, extinctions are higher in the female group (Table 2).

In contrast to apparently healthy patients (Table 1), the MM partition coefficient at 280/254 nm in the male group insignificantly increases (1.9%), while in the female group this coefficient decreases by 0. 7% compared to the youngest age group (40–49 years). It is possible to assume that the partition coefficient in the saliva of patients with lung cancer had initially high values even in the age group of 40–49 years, so there is an insignificant increase in the growth of endotoxemia phenomena. At the same time, in the group of apparently healthy people, an increase in the partition coefficient occurs due to age-related changes.

Generally, the nature of changes for both absolute values and the MM partition coefficient at 280/254 nm do not show any statistically significant gender-specific differences (Table 1 and Table 2). At the next research stage, the male group was only examined, as due to epidemiologic peculiarities, the lung cancer rate in men is much higher than in women. The research included a group of 337 cancer patients, consisting of 256 men and 81 women. Due to an inhomogeneous age distribution of study participants, it is seemingly impossible to make a correct statistical evaluation of studied parameters for women, and it is necessary to collect more observational records.

The evaluation has shown that MM levels in the saliva increase with age both under normal conditions (Table 1) and in the context of a cancer pathology (Table 2). At the same time, there is an increase in both fractions of an aromatic (280 nm) and non-aromatic origin (254 nm). The slope index for the trend line in the control group is 1.47–1.49 times as large as in the group of patients with lung cancer.

The evaluation of MM partition coefficient values at 280/254 nm has shown that in the control group, the MM fraction at 280 nm rises about 30% quicker than the MM fraction at 254 nm. This ensures a stable uniform growth in the MM level at 280/254 nm, while the average value remains at the level of 0.855 ± 0.030 units.

In the group of patients with lung cancer, the partition coefficient is almost the same with age. It is in the range of 0.977 ± 0.028 units. Thus, despite the fact that the dynamics of changes in individual MM fractions in different groups are practically the same, the partition coefficient is statistically much higher in the group of patients with lung cancer. The MM fraction at 254 nm is an integrated index for levels of UV-absorbing substances of low and average molecular weight, which, besides proteolysis products, include non-protein substances of normal and abnormal metabolism. The intensity of saliva UV absorption at 280 nm is mainly measured with available aromatic chromophores and its increase is due to a pileup of tryptophan- and tyrosine-containing peptides. It might be caused by a loss of aromatic amino acids in proteins because of oxidative modification and molecule fragmentation. A more active growth in the MM fraction at 254 nm in patients with lung cancer might mean more intensive catabolic processes and the stimulation of lipid peroxidation and immunogenesis.

**Figure 1:**
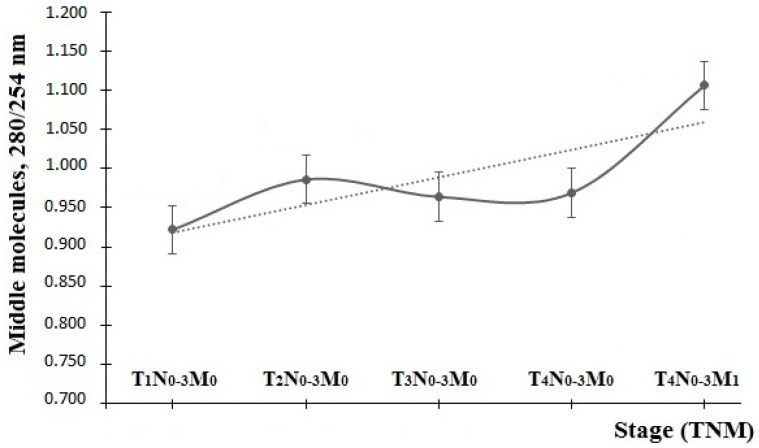
Dynamics of MM levels under normal conditions and in the context of cancer pathology.

The group of patients with lung cancer was examined for changes of the partition coefficient depending on the disease stage according to the TNM (Tumor, Nodus, Metastasis) system (Figure 1). It has been found that MM levels at 280/254 nm remain approximately unchanged up to the fourth stage inclusively, and amount to 0.927 ± 0.024 units. At the same time, they significantly increase in the case of available metastatic lesions up to 1.106 ± 0.017 units, which results from the proliferation of endotoxemia phenomena.

To confirm the hypothesis that the revealed changes are caused by an available oncologic disease, the next research stage included measurements of MM levels in the context of a non-malignant tumor. It has been shown that the MM partition coefficient at 280/254 nm for this group is 0.888 ± 0.021 units. These values are intermediate between the apparently healthy volunteers (0.855 ± 0.030 units) and patients with cancer pathology (0.977 ± 0.028 units). However, with age, the partition coefficient value decreases, reaching 0.937 ± 0.019, 0.895 ± 0.021, and 0.832 ± 0.017 units for the age groups of 40–49, 50–59, and 60–69 years, respectively.

Since the group of patients is not uniform, the MM partition coefficients at 280/254 nm were calculated for individual non-malignant lung diseases. Their values were 0.883 ± 0.016 units for hamartoma, 0.857 ± 0.019 units for pulmonary tuberculosis, and 0.892 ± 0.021 for sarcoidosis. Statistically significant differences between groups with non-malignant lung diseases were not found, but the degree of endotoxemia in all groups is lower than in the group with lung cancer. It should be accentuated that in the case of pulmonary tuberculosis, the partition coefficient corresponds to the value for the group of apparently healthy people, whereas absolute values of extinctions at wavelengths of 254 and 280 nm are substantially lower (0.190 ± 0.016 and 0.154 units ± 0.009 units, respectively). Despite the fact that the partition coefficients of hamartoma and sarcoidosis are lower than that of pulmonary tuberculosis, the absolute values of extinctions are higher. Thus, absorption at the wavelengths of 254 and 280 nm is 0.347 ± 0.016 and 0.312 ± 0.011 units for hamartoma; 0.428 ± 0.015 and 0.379 ± 0.018 for sarcoidosis, respectively. In this regard, for an integrated assessment of endotoxemia phenomena, it is necessary to consider both extinctions at the individual wavelengths and the calculated partition coefficient.

MM levels reflect the deposit rate for low-molecular-weight proteins, peptides, and degradation products of membrane lipids in the blood. Accordingly, it is an integrative index of body catabolic reactions and autointoxication [28]. In this regard, the MM partition coefficient at 280/254 nm might be used in dynamics to control endotoxemia phenomena while treating patients with cancer.

The revealed fact of the increased MM level is an indirect proof of the excessive generation of active oxygen metabolites superoxide radicals, and hydrogen dioxide [29]. When they interact with each other, the Haber-Weiss reaction generates hydroxyl radicals able to damage unsaturated fatty acids in phosphoglyceride structures of cell membranes and cell organoids [30]. Active oxygen metabolites influence the phospholipid base of membrane structures. Its high vulnerability depends on available phosphoglycerides, making the bulk of membrane structures, and arachidonic acid containing four double bonds, divided by CH_2_-groups. When active oxygen metabolites influence the latter, a hydrogen atom splits off, double bonds become conjugated and diene conjugates appear. Their further exposure to an attack by reactive oxygen metabolites leads to the formation of lipid hydroperoxides. As a result, the physico-chemical properties of membranes change: hydrophilic holes appear in the hydrophobic layer, which leads to a disruption in cell permeability and eventually to the cell damage. Hydroperoxides of fatty acids split, forming MDA and other substances, which react with thiobarbituric acid, producing colored compounds [30].

### 3.2. Measuring Products of Lipid Peroxidation

**Figure 2:**
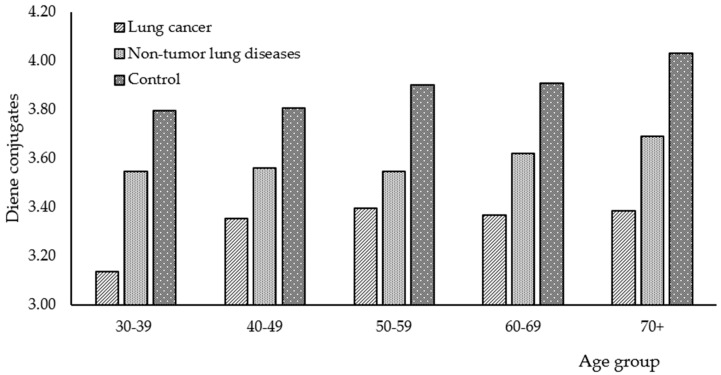
Levels of diene conjugates in saliva of patients with lung cancer, non-malignant lung diseases and of apparently healthy patients.

Products of lipid peroxidation are primary (diene conjugates) and secondary (triene conjugates and Schiff’s bases). In further research, individual products of lipid peroxidation were measured in the saliva of patients with lung cancer, non-malignant lung diseases, and apparently healthy people. We have found significant differences in the levels of the above-mentioned components between study groups (Figure 2).

The levels of diene conjugates in the saliva decrease by 8.5% when going from the control group to patients with non-malignant pulmonary pathology, and then, when going to patients with lung cancer (3.89, 3.59, and 3.33 units are average values for each group, respectively). The described changes are common for all age groups included in the study. Insufficient levels of lipid peroxidation products might result from the tumor tissue’s resistance to peroxide initiators and the modified functioning of the enzyme systems that regulate lipid peroxidation [31,32].

However, as for triene conjugates and Shiff bases, there is an inverse relationship: under normal conditions, levels of these products are much lower than in the case of lung diseases. Moreover, the differences between malignant and non-malignant diseases are not statistically significant (Table 3 and Table 4).

The trend is worth mentioning when, with age, levels of lipid peroxidation products increase in all study groups. Levels of Schiff bases in the group of patients with lung cancer is the only exception (Table 4). It seems that due to the age-specific decline in the metabolic rate, people from older age groups accumulate more secondary lipid peroxidation products. Shiff bases are end products in lipid peroxidation. They are irreversible cross-links between dialdehydes and free amino groups of membrane proteins and are indicative of the stage of cellular degenerative changes. On the other hand, the high level of Schiff bases might be considered an adaptive process, aimed at eliminating more toxic metabolites of lipid peroxidation from cells, i.e., diene conjugates and malondialdehyde.

**Figure 3:**
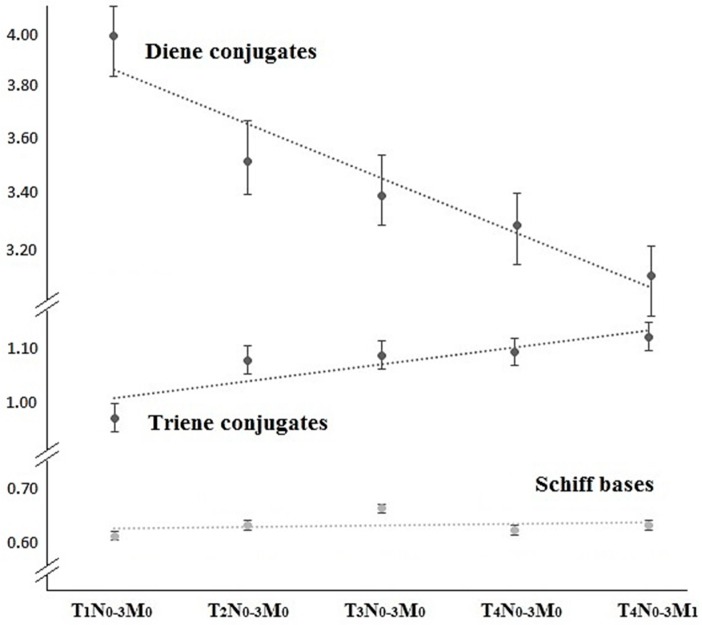
State of lipid peroxidation processes depending on the disease stage.

It is worth paying attention to the change in the activity of lipid peroxidation processes, depending on the disease stage (Figure 3). Thus, levels of Schiff bases remain at the stably high level compared to the control group (*p* < 0.001). Similarly, for triene conjugates, there is a slight increase in their levels when going from local to locally advanced disease stages.

As for diene conjugates, with disease progression, their levels decrease, reaching the minimal value of 3.09 units in metastatic lesions, which implies the highest inhibition of free radical processes, progressing as the tumor grows. At early stages of its development, a malignant tumor has an increased antioxidant activity, which helps to protect its cells from death. Although, as the tumor grows, there is a decrease in the activity of antioxidants and in the levels of lipid peroxidation products at the expense of increased levels of saturated fatty acids in cell membranes.

### 3.3. Measuring Malondialdehyde Levels

**Figure 4:**
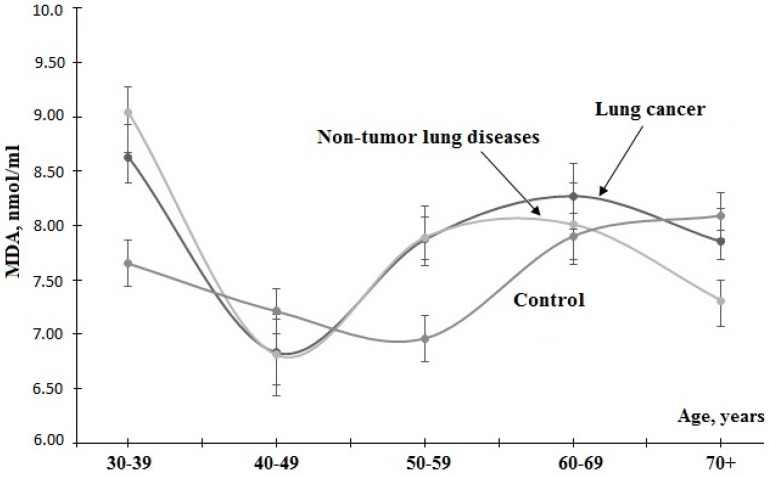
Dynamics of MDA levels depending on age.

To get a complete understanding of processes in endogenous intoxication and lipid peroxidation, it is necessary to consider all the stages. MDA is an end product of lipid peroxidation. The findings from the measured MDA for apparently healthy people and patients with lung cancer and non-malignant lung diseases are given in Figure 4. As can be seen, there is an ambiguous change in this parameter in the study groups (Figure 4).

MDA levels are significantly higher in the saliva of both apparently healthy patients and patients with malignant and non-malignant lung diseases in the age group of 30–39 years. This might be due to the high rate of metabolic processes, which shifts the equilibrium of lipid peroxidation towards the end product. It has been found that under normal conditions, MDA levels decrease with age, but after 60 years old, they increase up to an initial value and then reach a plateau. The average value of MDA levels in the group of apparently healthy volunteers amounted to 7.02 ± 0.25 nmol/mL. In the group of patients with lung diseases, the dynamics of changes in MDA levels is almost the same; there is the local minimum of the concentration corresponding to the age group of 40–49 years, and the local maximum for the age group of 60–69 years (Figure 4). The average MDA levels increase up to 7.76 ± 0.18 and 8.24 ± 0.12 nmol/mL for patients with benign and malignant diseases, respectively.

**Figure 5:**
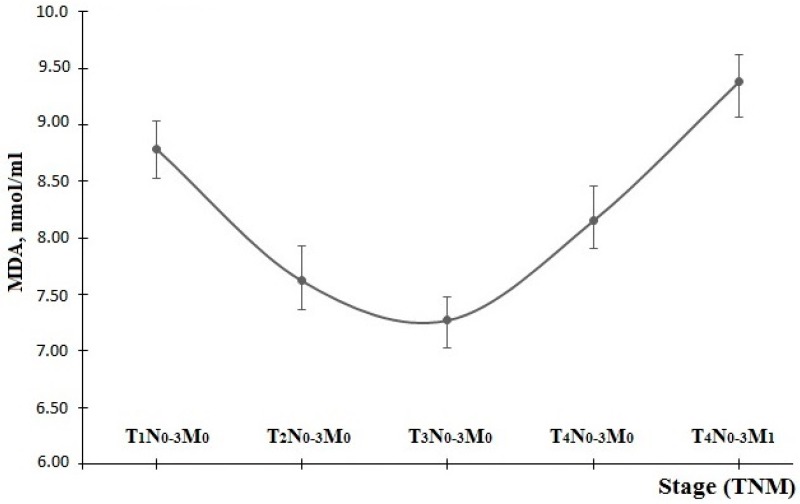
Dynamics of MDA level, depending on disease stage.

The dynamics of the MDA level depending on the disease stage show a gradual decrease from 8.79 ± 0.19 nmol/mL at the first stage to 7.27 ± 0.16 nmol/mL at the third stage. Then, there is an intensive growth in this parameter of up to 9.38 ± 0.2 nmol/mL in the context of metastatic lesions (Figure 5).

There is a large body of evidence showing that the increased MDA contents are not only evidence of intensified oxidative stress [33]. Being a toxic product, MDA contributes to endogenous intoxication of the body. The revealed correlations between the increased MDA contents and the degree of intoxication allow us to suggest the term “index of available reactive oxygen species”, considering MDA a marker of lipid peroxidation and tissue damage [33].

When comparing the dynamics of the MDA concentration and the MM partition coefficient at 280/254 nm, we can mention that changes in these parameters are antiphase (Figure 1 and Figure 5). The initial stage of tumor development corresponds to the high level of MDA and the low level of middle-molecular-weight toxins, which assumes severe damage in cell membrane lipids and a slow catabolic rate. In the course of tumor progression, the share of lipid hydroperoxides is decreasing, while the share of low-molecular-weight proteins and peptides is increasing, which assumes intensified endogenous intoxication up to the third stage. When going to the fourth stage of the disease, there is an increase in products of both lipid peroxidation and protein intoxication, which is especially evident in the case of tumor metastasis. This may be due to the growing necrosis in the tumor tissue.

## 4. Discussion

Disorders of the body’s antioxidant status play a crucial part in the pathogenesis of both tumors and complications resulting from their treatment, as they lead to toxic damage in the cell membrane and an aggravation of endogenous intoxication syndrome. It was found that the tumor tissue can pile up natural antioxidants, which leads to an inhibition of lipid peroxidation inside the tumor and decreased antioxidant defense in normal tissues. Such disorders in the oxidant-antioxidant balance cause damage in healthy tissues and contribute to the progression of endogenous intoxication, while at the same time stimulating tumor cell proliferation [34].

Numerous researchers have discussed endogenous intoxication and lipid peroxidation in the blood and saliva, but the process is only described with a pileup of the end product, MDA [19,23,25,26,35]. Thus, the research of the MDA level in the context of pre-cancerous changes and squamous cell cancer of the mouth shows that the MDA concentration increases by 80% when going from the control group to pre-cancerous changes, and by 183% when going to moderately differentiated squamous cell cancer [35]. Maximum differences were found between healthy people and patients with highly differentiated cancer of the oral cavity (213% compared to the control group). In the opposite direction, there is a decrease in glutathione, indicating the decreased activity of antioxidant enzymes. Similar results were obtained in studies of pre-cancerous changes in the oral cavity [19,23,25,26].

In our opinion, it is no less important to make a comprehensive assessment of endotoxemia processes by individual fractions of medium-molecular-weight toxins, which are a characteristic of catabolic processes and of a level of primary and secondary products in lipid peroxidation, which mark damage in the cell membrane.

## 5. Conclusions

Human saliva can be used as a biosubstrate to make comprehensive measurements for levels of endogenous intoxication and lipid peroxidation. The measured MDA as the end product of lipid peroxidation is less informative as it just helps to state the fact that this parameter increases in the context of an oncologic pathology. To get more information, it is necessary to measure individual fractions of middle-molecular-weight toxins, calculate the MM partition coefficient at 280/254 nm, and take into consideration levels of diene and triene conjugates and Shiff bases. This might be used to control endotoxemia phenomena in treatment of oncologic patients and to describe disorders of cell membrane structures.

The MM partition coefficient at 280/254 nm is an integrative index of catabolic reactions and body autointoxication. Normal values are 0.855 ± 0.030 units; in the context of a non-malignant lung disease, this value increases to 0.888 ± 0.021 units. In the context of lung cancer, this value increases up to 0.977 ± 0.028 units. The highest value is achieved in the case of regional and distant metastatic lesions.

We have revealed decreased levels of primary lipid peroxidation products (diene conjugates) in the context of lung cancer, with a simultaneous increase in levels of secondary products (triene conjugates, Shiff bases). This process is more evident when going from local to locally advanced disease stages. MDA is a marker of lipid peroxidation, but the nature of its change is non-linearly associated with the tumor progression.

The findings might be used to elaborate traditional aids for diagnostics and forecasting of the disease state, as well as in treatment monitoring, etc.

## Figures and Tables

**Table 1:** MM levels under normal conditions depending on gender and age.

Age, Years	Gender	MM at 254 nm	MM at 280 nm	MM at 280/254 nm
40–49	M	0.327 ± 0.024	0.265 ± 0.020	0.810 ± 0.021
	F	0.310 ± 0.022	0.265 ± 0.025	0.855 ± 0.019
50–59	M	0.364 ± 0.021	0.295 ± 0.024	0.810 ± 0.022
	F	0.315 ± 0.018	0.264 ± 0.021	0.838 ± 0.021
60–69	M	0.361 ± 0.022	0.325 ± 0.023	0.900 ± 0.019
		–	*p* = 0.046	*p* = 0.048
	F	0.316 ± 0.020	0.261 ± 0.018	0.825 ± 0.024
Over 70	M	0.451 ± 0.021	0.417 ± 0.019	0.924 ± 0.018
		*p* = 0.037	*p* = 0.026	*p* = 0.002
	F	0.361 ± 0.018	0.317 ± 0.023	0.878 ± 0.025
		*p* = 0.010	*p* = 0.003	–

Note: *p—*statistically significant differences compared to the age group of 40–49.

**Table 2:** MM levels depending on gender and age in patients with lung cancer.

Age, Years	Gender	MM at 254 nm	MM at 280 nm	MM at 280/254 nm
40–49	M	0.240 ± 0.020	0.203 ± 0.017	0.935 ± 0.026
	F	0.249 ± 0.019	0.212 ± 0.023	0.914 ± 0.026
50–59	M	0.282 ± 0.022	0.257 ± 0.026	1.017 ± 0.022
		–	–	*p* = 0.037
	F	0.275 ± 0.018	0.254 ± 0.024	0.995 ± 0.023
60–69	M	0.314 ± 0.017	0.290 ± 0.023	1.010 ± 0.029
		*p* = 0.022	*p* = 0.009	*p* = 0.045
	F	0.314 ± 0.020	0.284 ± 0.023	0.985 ± 0.021
Over 70	M	0.313 ± 0.019	0.301 ± 0.024	0.953 ± 0.018
		*p* = 0.025	*p* < 0.001	–
	F	0.328 ± 0.021	0.292 ± 0.021	0.908 ± 0.022
		*p* < 0.001	*p* = 0.012	–

Note: *p—*statistically significant differences compared to the age group of 40–49.

**Table 3:** Levels of triene conjugates in saliva of patients with lung cancer, non-malignant lung diseases and of apparently healthy patients (control).

Age, Years	Control (1)	Non-Malignant Lung Disease (2)	Lung Cancer (3)
30–39	0.859 ± 0.014	1.033 ± 0.011	1.089 ± 0.021
	–	–	*p*_2_ = 0.002
40–49	0.885 ± 0.011	1.057 ± 0.016	1.084 ± 0.019
	–	*p*_2_ = 0.023	*p*_2_ < 0.001
50–59	0.886 ± 0.015	1.058 ± 0.015	1.083 ± 0.014
	*p*_1_ = 0.009	*p*_2_ < 0.001	*p*_2_ < 0.001
60–69	0.899 ± 0.021	1.075 ± 0.015	1.088 ± 0.016
	*p*_1_ = 0.004	*p*_1_ = 0.045, *p*_2_ = 0.004	*p*_2_ < 0.001
Over 70	0.953 ± 0.015	NA	1.128 ± 0.009
	*p*_1_ < 0.001	–	*p*_2_ < 0.001

Note: *p*_1_*—*statistically significant differences compared to the age group of 30–39; *p*_2_*—*statistically significant differences compared to the control group.

**Table 4:** Levels of Shiff bases in saliva of patients with lung cancer, non-malignant lung diseases and of apparently healthy patients (control).

Age, Years	Control (1)	Non-Malignant Lung Disease (2)	Lung Cancer (3)
30–39	0.515 ± 0.014	0.584 ± 0.018	0.632 ± 0.017
	–	–	*p*_2_ = 0.001
40–49	0.529 ± 0.011	0.590 ± 0.017	0.631 ± 0.020
	–	–	*p*_2_ = 0.038
50–59	0.534 ± 0.016	0.621 ± 0.021	0.629 ± 0.015
	*p*_1_ = 0.005	*p*_2_ = 0.004	*p*_2_ < 0.001
60–69	0.557 ± 0.010	0.623 ± 0.017	0.625 ± 0.010
	*p*_1_ = 0.002	*p*_1_ = 0.038, *p*_2_ = 0.014	*p*_2_ = 0.007
Over 70	0.637 ± 0.020	NA	0.611 ± 0.010
	*p*_1_ < 0.001	–	–

Note: *p*_1_—statistically significant differences compared to the age group of 30–39; *p*_2_—statistically significant differences compared to the control group.
